# Key Factors of Opening Gated Community in Urban Area: A Case Study of China

**DOI:** 10.3390/ijerph18073401

**Published:** 2021-03-25

**Authors:** Zezhou Wu, Lu Yang, Kexi Xu, Jinming Zhang, Maxwell Fordjour Antwi-Afari

**Affiliations:** 1 Sino-Australia Joint Research Center in BIM and Smart Construction, Shenzhen University, Shenzhen 518061, China; wuzezhou@szu.edu.cn (Z.W.); yanglu2018@email.szu.edu.cn (L.Y.); 2 School of Public Administration, Zhejiang University of Finance and Economics, Hangzhou 310018, China; 3 School of Political Studies, Nanjing Agricultural University, Nanjing 210095, China; zjming@njau.edu.cn; 4 Department of Civil Engineering, College of Engineering and Physical Sciences, Aston University, Birmingham B4 7ET, UK; m.antwiafari@aston.ac.uk

**Keywords:** gated community, logistic regression, willingness, key factors

## Abstract

Gated communities are the most popular residential pattern in the urban areas of China. However, along with the increasing population density in urban areas, this pattern may have negative influences on people’s daily lives, such as traffic jams. To avoid the negative influences, the government has encouraged residents to open their gated communities; however, few positive actions have been taken. With this background, this study aims to explore the key factors in residents’ willingness to open their gated communities. To start with, a total of 26 potential factors were identified based on a comprehensive literature review. Then, a questionnaire was designed and distributed to collect empirical data. Furthermore, logistic regression was employed to analyze the collected data. Based on the derived results, it was revealed that concerns are different between male and female residents. Male residents regarded “community safety” and “property management” as having a significant impact on their decision to open a gated community, while female residents paid more attention to the factor of “proprietary equity”. The results of this study could provide valuable references that enable the government to better understand residents’ underlying concerns and to make relevant policy decisions.

## 1. Introduction

In recent decades, China has been experiencing a rapid urbanization process [1,2,3,4]. Numerous residential communities have been developed in urban areas, and gated communities are the most popular residential pattern. A gated community usually encloses the buildings and their public support facilities in a certain geographical scope and has a clear boundary with walls and has several imports and exports [5]. In China, a gated community usually covers an area of 12 to 20 hectares and contains 2000 to 3000 households [6]. 

During the long period of China’s history, the gated community has been regarded as the main residential pattern as it has several advantages, such as guaranteeing people’s living safety, privacy and living environment [7]. In the 1980s, due to the economic and social circumstances, the gated community even became the template for developing domestic residential buildings in China [8]. In recent decades, with the rapid development of China’s real-estate market, gated communities have been increasingly favored by developers [9,10,11]. With this background, living in a gated community has gradually become a symbol of people’s daily life [12]. 

Nevertheless, with the rapid urbanization process and the drastic increasing number of gated communities in recent decade, a number of urban problems has emerged in many cities, such as urban traffic congestion, public facilities division and neighborhood environmental quality [13,14,15]. In order to solve the emerging urban problems, in 2016 the central government of China promulgated a top-down policy which instructed that no gated community be allowed to be built in the future and the gated communities that had been built should be gradually opened up [16]. This policy has given rise to intense arguments in both the society and academia since its first announcement. Various voices on the pro and con sides of this policy appeared. According to investigations conducted by the authoritative media, most residents held negative attitudes to opening their closed communities [17]. Nevertheless, the central government seemed very persistent at that time [18]. However, after three years of the policy being promulgated, it is reported that the number of gated communities has not decreased and the newly built communities still conform to the pattern of closed communities. In addition, the government is also no longer insisting on the promotion of this policy at present. It seems that the residents’ unwillingness to open their gated communities eventually influenced the government’s decision. Thus, there is an interesting question: What are the key factors that influence residents’ willingness to open their gated communities?

Bearing this research question in mind, this study aims to identify the most significant factors that influence residents’ willingness to open their closed communities. A comprehensive review was conducted to collect the suggested advantages and disadvantages of gated communities from the existing literature. Then, follow-up interviews were carried out to further formulate a formal questionnaire. Based on the data collected through questionnaire surveys, the research findings are presented and discussed at the end of this paper.

## 2. Literature Review

In the existing literature, researchers have explored gated communities from different perspectives. For example, Liao et al. [19] employed a structure-agency approach to investigate the role of urban planners in the production of gated communities in China. It was revealed that the values of urban planners and the structural factors that influence their preferences have a certain influence on the formation of gated communities. Different from open communities, a gated community has closed management which prohibits the access of outside people and vehicles. Using principal component analysis, Ehwi et al. [20] explored the reasons for the growing number of gated communities around the world from a land management perspective. Results showed that an obvious advantage of a gated community is the security assurance, which is echoed with the research findings of Lo and Wang [8]. Studies further showed that the crime rate in non-gated communities is 25 times higher than that in gated communities because gated communities provide better security for residents [21]. Nevertheless, as the economy and society have changed, the effects of gated communities have been discussed from both positive and negative perspectives.

Various studies have been conducted to discuss the side effects of gated communities. For example, Sun et al. [22] recorded the complete actual walking routes of 3637 metro users from station exits to their destinations, revealing that the shortest path was cut off because of gated communities. From the perspective of the relationship between human beings and the environment, Zhang et al. [23] explored the influence of the existence of gated communities on the allocation of green resources in cities by revisiting an increasingly popular Gaussian-two-step floating catchment area model. Ozdemir and Turkseven Dogrusoy [24] further argued that gated communities undermine the relationship between humans and the environment and cause significant handicaps in terms of public life and the sustainability of open public spaces.

Different community patterns can also have impacts concerning social perspectives. As income inequality has become more and more visible in big cities, Lestari Olivia et al. [25] argued that a mixed-income gated community contradicts the objective of the balanced housing policy because of the occurrence of social interactions among different economic strata. Roitman and Recio [26] further confirmed that inequality in residents’ income intensifies the formation of gated communities. Tandogan [27] compared children’s outdoor games between gated communities and non-gated residential neighborhoods. It was observed that children living in non-gated residential communities spend more time playing outdoors when compared to children living in gated communities. Furthermore, Akgun Gultekin and Unlu [28] claimed that the gated-community pattern can bring perceived stress to the neighbors.

Researchers also investigated the barriers to opening the gated communities from different perspectives, such as property law, privacy and employment pressure [29]. However, few studies have investigated the underlying reasons why people are not willing to open gated communities. Thus, this study aims to explore the factors affecting residents’ willingness to opening gated community.

## 3. Research Methodology

### 3.1. Identification of Potential Factors

To identify the potential factors that affect the opening of gated community, a literature review was conducted. Through the literature review, a total of 26 potential factors were identified, as shown in Table 1. According to their literal meanings, the identified factors were categorized into seven groups: Community area (CA), community environment (CE), community safety (CS), city traffic (CT), proprietary equity (PE), property management (PM) and social development (SD).

### 3.2. Data Collection

Based on the identified factors listed in Table 1, a preliminary questionnaire was initially designed to collect people’s perceptions. The preliminary questionnaire was further distributed to five experts to solicit their opinions on the comprehensiveness of the influential factors. By considering their suggestions, a formal questionnaire was finalized, as shown in Appendix A.

In the designed questionnaire, the classical five-point Likert scale was used to measure respondents’ agreement with the identified factors, in which “1” refers to “strongly disagree”, “2” represents “disagree”, “3” means “neutral”, “4” stands for “agree” and “5” is “strongly agree”. The “neutral” option indicates that the respondent does not have an obvious tendency concerning both gated and opened patterns.

The questionnaire was distributed and collected through a field investigation method. Several rounds of field investigations were conducted in Shenzhen during the period from 6 January 2019 to 26 March 2019. Respondents were randomly selected on streets based on their willingness to fill out the questionnaires. Finally, a total of 312 questionnaires were collected for further data analysis.

### 3.3. Logistic Regression Model (LRM)

SPSS (Statistical Product and Service Solutions) software was used to test the reliability of the collected data. The coefficient of Cronbach’s α was derived to judge the reliability. Chan et al. [44] suggested that the following outcomes are commonly accepted: For the value of “α > 0.9”, excellent; “α > 0.8”, good; “α > 0.7”, acceptable; “α > 0.6”, questionable; “α > 0.5”, poor; and “α < 0.5”, unacceptable. In this survey, the “α” value was 0.825, which suggests the data were reliable. Logistic regression analysis was further employed to assess the relationship between various factors and the willingness of residents to open their gated community.

The maximum likelihood (ML) method was used to estimate the parameters in the logistic regression models after transforming the dependent variable into a logit variable [45]. As such, logistic regression estimates the probability of a certain event occurring by calculating changes in the logarithm of the dependent variable rather than changes in the dependent variable itself, as ordinary least square (OLS) regression does [46].

## 4. Results and Discussions

### 4.1. Descriptive Statistical Analysis

Table 2 shows the background information of the respondents. As can be seen in Table 2, among the respondents, 44.23% were male and 55.77% were female. In general, a majority of the respondents were under the age of 40, and 95% of the respondents had a bachelor’s degree or above. This is because Shenzhen is a vibrant city which attracts a huge number of excellent and young people from the whole country [47]. In addition, because the housing price in Shenzhen is very high, a majority of the young people have no house of their own, it can be seen that 42.31% of the respondents were tenants. In terms of the residential pattern, most of the communities were gated or semi-gated, representing 77.56%. A majority of the respondents, 66.03%, did not own private cars. As for whether to open the gated community they may live in, 15.38% of the respondents supported it, 48.72% were neutral and 35.90% opposed it. However, as for whether to open the gated community inhabited by others, people’s attitudes changed, with 19.87% of respondents expressing support, 63.46% maintaining a neutral attitude and 16.67% opposed.

### 4.2. Logistic Regression

In this study, logistic regression was employed to investigate the key factors of opening gated communities. Firstly, the collected data of all respondents were analyzed using logistic regression. Results showed that the *p* value derived from the Hosmer–Lemeshow test was less than 0.05, which indicated that the model does not adequately fit the data. The respondents were divided into different groups according to personal information. Results reveal that, except for the gender groups, the *p* values of the Hosmer–Lemeshow tests of other groups were less than 0.05; thus, two independent logistic regression models (i.e., male group and female group) were used to test the identified factors. Table 3 shows the fitting degree of the logistic regression models. The *p* values of the Hosmer–Lemeshow tests were 2.869 and 8.229, respectively, indicating good data fit in the two models.

Table 4 shows the logistic regression results of Model 1 (male respondents) and Model 2 (female respondents). According to the comparison results, it can be concluded that male residents are more concerned about community safety and property management, while female residents focus on proprietary equity. Meanwhile, it should be noted that both male and female residents care about community environment changes and city traffic improvement; however, their concerns were different. 

### 4.3. Discussions

To test the empirical results, three professionals who are experienced in this field were invited to provide their comments. The three invited interviewees included a government officer, a real estate developer and a scholar. The government officer advocated opening gated communities. From her perspective, the advantages of this measure outweigh the disadvantages; more specifically, the opening of gated communities can effectively solve social problems such as traffic jams. Unlike the government officer, the real estate developer was more concerned about his own economic benefits. In the current situation, gated communities are favored by the public, so the developer believed developing gated communities could attract more customers and improve profits. From the scholar’s point of view, whether to open the gated community should be considered according to the local conditions. This measure only makes sense under the premise that the opening of a gated community could bring more benefits.

It is not surprising that community safety is an important factor that influences residents’ willingness to open gated communities. During the process of national development, social stability is an essential prerequisite. Fences and walls can physically isolate a community’s residents from the public; thus, the gated-community pattern can prevent potential crimes to a large extent. In this regard, residents prefer to live in a gated community, and the developer generally adopts the gated-community pattern for new residential buildings to improve sales. However, the governmental officer held the opinion that, with economic and social development, crime rates in urban areas have decreased to a large extent. In these circumstances, the function of guaranteeing safety should not be obvious in the future. However, the officer also agreed that it takes time for the public to become aware of such changes. In previous studies, the relationship between safety and gated communities was investigated; however, the research findings showed some unexpected conclusions. For example, Breetzke et al. [48] found that gated communities are associated with increased levels of burglary in South Africa. In addition, Tanulku [33] conducted two cases studies in Turkey and found that gated communities could lead to new forms of danger.

In addition to community safety, proprietary equity is also emphasized when considering opening gated communities. In China, housing is the main asset of the Chinese people. In general, communities with high housing prices have more recreation facilities and prettier garden landscapes. The residents living in such communities are unwilling to open their gated community because they believe that they have paid for these privileges and they do not want to share the facilities and landscapes with the public. In these circumstances, it is understandable that gated communities are preferred. This phenomenon was also observed by the invited academic scholar, and he suggested that this problem may be solved along with the social and economic development which will bring more facilities and more beautiful landscapes to public areas. This research finding is echoed by Salah and Ayad [49]. They conducted a study to investigate the reasons for residents’ preference for gated communities in the city of Alexandria. The results showed that people preferred living in gated communities for certain values.

Property management is another critical aspect when residents consider opening their gated communities. This aspect is mainly focused on the economic perspective. Opening a gated community can reduce property management fees and increase the income channels of property management companies. However, property management is more difficult after a community is opened. Thus, residents are ambivalent to opening their gated communities from this perspective. This is also an important issue which requires a series of comprehensive and thoughtful guarantee measures to remove people’s concerns. Soyeh, et al. [50] argued that property prices and rent charges in gated communities are much higher than those in non-gated communities in Ghana.

The social development aspect was found to be insignificant based on the investigation results. From a historical perspective, the gated-community pattern has been there for thousands of years. In the ancient times, gated communities had the function of both guaranteeing residents’ safety and strengthening social control. In addition, China’s traditional culture emphasizes distance and hierarchy among people. In this context, even in modern times, a neighborhood’s impact on housing segregation attracts little attention, and ordinary citizens are more concerned with protecting themselves than with social interaction.

## 5. Conclusions

This study investigated the key factors of opening gated communities in the urban areas of China. A total of 312 questionnaires were collected and the logistic regression model was used for data analysis. Results showed that community safety, proprietary equity and property management were the three key factors that influence residents’ willingness to open their gated communities. In the meantime, it was found that residents care less about the social development aspect of opening a gated community. From the survey results, the number of residents who are unwilling to open their own communities was much more than the number of residents who are willing to. This result is mainly because the residents have paid too much money for their residence, and they do not want to share the privileges with the public. Based on the above research findings, this study suggested that it may be inappropriate to compulsorily require the gated communities to be opened immediately. In other words, the government should solve the problems that the residents care about the most before the compulsory requirement of opening gated communities.

This study has made contributions to the existing knowledge, as there was no such study in Shenzhen; the research findings could aid relevant policy makers in formulating more effective and appropriate measures to design the city better. However, this research also has some limitations. For example, the majority of the respondents was under the age of 40. It is recommended that in future research the questionnaire be distributed to more respondents, covering all age groups. In addition, the vignette approach is suggested as it can be used to deal with sensitive topics and to capture the context of decision making.

## Figures and Tables

**Table 1:** Factors affecting the opening of gated community.

Category	Code	Factors	Impact	Source
Community area (CA)	CA1	Increase public areas	Positive	[30]
	CA2	Improve the utilization rate of the community area	Positive	[30]
Community environment (CE)	CE1	Increase the exposure to vehicle exhaust	Negative	[31]
	CE2	Increase the risk of noise disturbance to residents	Negative	[31]
	CE3	Increase the garbage pollution to community	Negative	[31]
	CE4	Increase the number of posted ads in the community	Negative	[31]
	CE5	Increase disorderly parking in the community	Negative	[32]
Community safety (CS)	CS1	Increase the difficulty of protecting private properties	Negative	[33]
	CS2	Increase the possibility of traffic accidents in the community	Negative	[34]
	CS3	Increase the risk of owners’ privacy invasion	Negative	[35]
	CS4	Reduce residents’ personal safety in the community	Negative	[35]
City traffic (CT)	CT1	Reduce the occurrence of traffic jams	Positive	[36]
	CT2	Increase non-motor vehicle flows on the branch roads	Positive	[37]
	CT3	Reduce the time of traffic congestion	Positive	[38]
Proprietary equity (PE)	PE1	Reduce residents’ utilization rate of community facilities	Negative	[39]
	PE2	Increase the risk of damage to the community facilities	Negative	[39]
	PE3	Decrease owners’ equity in the community	Negative	[35]
Property management (PM)	PM1	Reduce property management fees	Positive	[40]
	PM2	Increase income channels of property management companies	Positive	[40]
	PM3	Increase the property maintenance costs	Negative	[40]
	PM4	Increase the difficulty of property management	Negative	[40]
Social development (SD)	SD1	Weaken the division of social classes	Positive	[41]
	SD2	Stimulate the vitality of the community atmosphere	Positive	[41]
	SD3	Increase the inclusiveness of a city	Positive	[42]
	SD4	Reduce the residents’ sense of ownership	Negative	[43]
	SD5	Reduce the residents’ sense of respect	Negative	[43]

**Table 2:** Basic information of respondents.

Item	Category	Number	Percentage
Gender	Male	138	44.23%
	Female	174	55.77%
Age	Under 20	20	6.41%
	20~29	209	66.99%
	30~39	71	22.76%
	40~49	6	1.92%
	50 and above	6	1.92%
Education	PhD	14	1.28%
	Master	185	34.94%
	Bachelor	109	59.29%
	Senior high school or below	4	4.49%
Identity	House owner	171	54.81%
	House tenant	132	42.31%
	Property manager	2	0.64%
	Government officer	7	2.24%
Residential pattern	Gated community	102	32.69%
	Semi-gated community	140	44.87%
	Open community	70	22.44%
Private car	With a private car	106	33.97%
	Without a private car	206	66.03%
Willingness to open their own gated community	Agree	48	15.38%
	Neutral	152	48.72%
	Disagree	112	35.90%
Willingness to open others’ gated community	Agree	62	19.87%
	Neutral	198	63.46%
	Disagree	52	16.67%

**Table 3:** Goodness of fit measures.

Model Fitting Statistics	Model 1 Male Respondents	Model 2 Female Respondents
χ2	65.89 (*p* = 0.000)	55.397 (*p* = 0.001)
−2Log likelihood	71.243	72.770
Hosmer-Lemeshow	2.869 (*p* = 0.942)	8.229 (*p* = 0.411)
Total sample	138	174

**Table 4:** Logistic regression analysis of Models 1 and 2.

Code	Model 1 Male Respondents			Model 2 Female Respondents		
	B	OR(Exp(B))	Sig.	B	OR(Exp(B))	Sig.
CA1	−0.077	0.926	0.901	0.091	1.095	0.876
CA2	1.699	5.47	0.005 ***	−1.297	0.273	0.065 *
CE1	−2.274	0.103	0.041 **	−0.101	0.904	0.899
CE2	−0.388	0.678	0.603	2.265	9.63	0.063 *
CE3	0.621	1.861	0.478	−1.281	0.278	0.179
CE4	−0.771	0.463	0.349	−1.196	0.302	0.078 *
CE5	1.392	4.023	0.124	1.099	3.002	0.168
CS1	1.451	4.265	0.01 ***	0.589	1.802	0.172
CS2	0.702	2.017	0.33	0.299	1.349	0.492
CS3	−1.235	0.291	0.041 **	0.316	1.371	0.634
CS4	1.965	7.138	0.005 ***	−0.456	0.634	0.486
CT1	−2.106	0.122	0.004 ***	0.822	2.276	0.234
CT2	0.457	1.58	0.368	−1.088	0.337	0.035 **
CT3	−0.597	0.55	0.329	1.204	3.332	0.079 *
PE1	0.616	1.852	0.167	−1.423	0.241	0.028 **
PE2	−0.054	0.948	0.943	−1.814	0.163	0.012 **
PE3	−0.015	0.985	0.981	1.547	4.697	0.042 **
PM1	−2.003	0.135	0.004 ***	−0.047	0.954	0.912
PM2	1.15	3.158	0.012 **	−0.113	0.893	0.801
PM3	1.056	2.874	0.029 **	−1.015	0.363	0.037 **
PM4	−0.842	0.431	0.171	0.862	2.367	0.16
SD1	0.756	2.129	0.073 *	−0.675	0.509	0.15
SD2	−0.853	0.426	0.182	−1.436	0.238	0.108
SD3	0.282	1.326	0.716	0.512	1.669	0.543
SD4	−0.143	0.867	0.822	−1.143	0.319	0.135
SD5	−0.462	0.63	0.45	0.771	2.163	0.207

Note: * Significant at 10% level; ** Significant at 5% level; *** Significant at 1% level.

## Appendix A. Questionnaire

### Appendix A.1. Part I: Basic Information

1. Your gender:
  ☐ Male   ☐ Female
2. Your age:
  ☐ Under 20   ☐ 20~29   ☐ 30~39   ☐ 40~49   ☐ 50 and above
3. Your education:
  ☐ PhD   ☐ Master   ☐ Bachelor   ☐ Senior high school or below
4. Your identity:
  ☐ House owner   ☐ House tenant   ☐ Property manager   ☐ Government officer
5. Residential pattern of your community:
  ☐ Gated community   ☐ Semi-closed and semi-open community    ☐ Open community
6. Do you have a private car:
  ☐ Yes   ☐ No
7. Willingness to open your own community:
  ☐ Agree   ☐ Neutral   ☐ Disagree
8. Willingness to open others’ community:
  ☐ Agree   ☐ Neutral   ☐ Disagree

### Appendix A.2. Part II: Evaluation of Affecting Factors

The following factors are regarded as potential influences of opening gated communities, please use 1~5 to indicate your agreement of these statements (1-Strongly disagree; 2-Disagree; 3-Neutral; 4-Agree; 5-Strongly agree).

**No.**	**Factors**	**1**	**2**	**3**	**4**	**5**
1	Increase the public areas	☐	☐	☐	☐	☐
2	Improve the utilization rate of the community area	☐	☐	☐	☐	☐
3	Reduce the occurrence of traffic jams	☐	☐	☐	☐	☐
4	Increase the non-motor vehicle flows on the branch roads	☐	☐	☐	☐	☐
5	Reduce the time of traffic congestion	☐	☐	☐	☐	☐
6	Reduce property management fees for owners	☐	☐	☐	☐	☐
7	Increase income channels of property management companies	☐	☐	☐	☐	☐
8	Weaken the division of social classes	☐	☐	☐	☐	☐
9	Stimulate the vitality of the community atmosphere	☐	☐	☐	☐	☐
10	Increase the inclusiveness of a city	☐	☐	☐	☐	☐
11	Increase the exposure to vehicle exhaust	☐	☐	☐	☐	☐
12	Increase the risk of noise disturbance to residents	☐	☐	☐	☐	☐
13	Increase the garbage pollution to community	☐	☐	☐	☐	☐
14	Increase the number of posted ads in the community	☐	☐	☐	☐	☐
15	Increase the disorderly parking in the community	☐	☐	☐	☐	☐
16	Increase the difficulty of protecting private properties	☐	☐	☐	☐	☐
17	Increase the possibility of traffic accidents in the community	☐	☐	☐	☐	☐
18	Increase the risk of owners’ privacy invasion	☐	☐	☐	☐	☐
19	Reduce residents’ personal safety in the community	☐	☐	☐	☐	☐
20	Reduce the residents’ utilization rate of community facilities	☐	☐	☐	☐	☐
21	Increase the risk of damage to the community facilities	☐	☐	☐	☐	☐
22	Decrease owner’s equity in the community	☐	☐	☐	☐	☐
23	Increase the property maintenance costs	☐	☐	☐	☐	☐
24	Increase the difficulty of property management	☐	☐	☐	☐	☐
25	Reduce the residents’ senses of ownership	☐	☐	☐	☐	☐
26	Reduce the residents’ senses of respect	☐	☐	☐	☐	☐
